# Dose‐ rather than fluence‐averaged LET should be used as a single‐parameter descriptor of proton beam quality for radiochromic film dosimetry

**DOI:** 10.1002/mp.14097

**Published:** 2020-03-13

**Authors:** Andreas Franz Resch, Paul David Heyes, Hermann Fuchs, Niels Bassler, Dietmar Georg, Hugo Palmans

**Affiliations:** ^1^ Division Medical Radiation Physics Department of Radiotherapy Christian Doppler Laboratory for Medical Radiation Research for Radiation Oncology Medical University of Vienna/AKH Wien Währinger Gürtel 18‐20 1090 Vienna Austria; ^2^ Medical Radiation Physics Department of Physics Stockholm University Stockholm Sweden; ^3^ Department of Oncology and Pathology Medical Radiation Physics Karolinska Institutet Stockholm Sweden; ^4^ Department of Experimental Clinical Oncology Aarhus University Hospital Aarhus Denmark; ^5^ MedAustron Ion Therapy Centre/EBG MedAustron Marie‐Curie‐Straße 5 2700 Wiener Neustadt Austria; ^6^ Medical Radiation Science National Physical Laboratory Hampton Road TW11 0LW Teddington United Kingdom

**Keywords:** beam quality correction, LET quenching, linear energy transfer, Monte Carlo simulations, proton beam therapy, radiochromic film dosimetry

## Abstract

**Purpose:**

The dose response of Gafchromic EBT3 films exposed to proton beams depends on the dose, and additionally on the beam quality, which is often quantified with the linear energy transfer (LET) and, hence, also referred to as LET quenching. Fundamentally different methods to determine correction factors for this LET quenching effect have been reported in literature and a new method using the local proton fluence distribution differential in LET is presented. This method was exploited to investigate whether a more practical correction based on the dose‐ or fluence‐averaged LET is feasible in a variety of clinically possible beam arrangements.

**Methods:**

The relative effectiveness (RE) was characterized within a high LET spread‐out Bragg peak (SOBP) in water made up by the six lowest available energies (62.4–67.5 MeV, configuration “$b_{1}$”) resulting in one of the highest clinically feasible dose‐averaged LET distributions. Additionally, two beams were measured where a low LET proton beam (252.7 MeV) was superimposed on “$b_{1}$”, which contributed either 50% of the initial particle fluence or 50% of the dose in the SOBP, referred to as configuration “$b_{2}$” and “$b_{3}$,” respectively. The proton LET spectrum was simulated with GATE/Geant4 at all measurement positions. The net optical density change differential in LET was integrated over the local proton spectrum to calculate the net optical density and therefrom the beam quality correction factor. The LET dependence of the film response was accounted for by an LET dependence of one of the three parameters in the calibration function and was determined from inverse optimization using measurement “$b_{1}$.” This method was then validated on the measurements of “$b_{2}$” and “$b_{3}$” and subsequently used to calculate the RE at 900 positions in nine clinically relevant beams. The extrapolated RE set was used to derive a simple linear correction function based on dose‐averaged LET ($L_{d}$) and verify the validity in all points of the comprehensive RE set.

**Results:**

The uncorrected film dose deviated up to 26% from the reference dose, whereas the corrected film dose agreed within 3% in all three beams in water (“$b_{1}$”, “$b_{2}$” and “$b_{3}$”). The LET dependence of the calibration function started to strongly increase around 5 keV/μm and flatten out around 30 keV/μm. All REs calculated from the proton fluence in the nine simulated beams could be approximated with a linear function of dose‐averaged LET (RE = 1.0258−0.0211 μm/keV $L_{d}$). However, no functional relationship of RE‐ and fluence‐averaged LET could be found encompassing all beam energies and modulations.

**Conclusions:**

The film quenching was found to be nonlinear as a function of proton LET as well as of the dose‐averaged LET. However, the linear relation of RE on dose‐averaged LET was a good approximation in all cases. In contrast to dose‐averaged LET, fluence‐averaged LET could not describe the RE when multiple beams were applied.

## 1 INTRODUCTION

Gafchromic EBT3 films are widely used dosimeters in photon as well as light ion beam therapy centers mainly because of their high spatial resolution and their near tissue equivalence. The active volume of EBT3 films and their precursors, EBT and EBT2, contain active lithium pentacosa‐10, 12‐diynoate (LiPCDA),^1^ a monomer, which polymerizes when exposed to ionizing radiation. The polymerization results in a darkening of the film, which is utilized to determine the absorbed dose. In an ideal detector with infinite monomer density, the number of polymers would linearly increase with absorbed dose, *D*. For EBT3 films this is approximately the case up to roughly 1 Gy. The simple assumption that a differential change in number of monomers is proportional to the available monomers results in an exponential relation of the number of formed polymers to absorbed dose.^2^, ^3^ Based on an idea ranging back to the 1960s^4^ the bimolecular model was proposed for EBT2 films^5^ assuming that two monomers need to interact to form a polymer. Several other models have been used in literature and there is no clear evidence of one being superior to the others.^6^, ^7^, ^8^ In a general way those models relate the net optical density, $o_{Q}$, to absorbed dose to water, $D_{w}$, given a set of parameters, ${\vec{p}}_{Q}$, which are valid at a certain beam quality, *Q*:

(1) $$o_{Q}=f\left(D_{w}|{\vec{p}}_{Q}\right).$$

Beam quality specific parameters, ${\vec{p}}_{Q}$, are obtained during calibration at a reference beam quality, $Q_{0}$. To avoid quenching, films are typically calibrated in a low LET beam, namely in a photon beam or in the entrance plateau of a high‐energy proton beam. By inverting Eq. (1) one can determine the dose from a measured net optical density in the clinically relevant dose range. However, the dose obtained in this manner is incorrect, if films are exposed to proton beams with a considerably different beam quality. Up to 20% underestimation of the dose (at the distal edge of a Bragg peak) was reported for proton beams.^5^, ^9^, ^10^ Such an underresponse was not only reported for ion beams, but also for low‐energy photons in the kilovoltage range with a similar up to 20% effect at 70 kVp.^11^ This energy dependence when exposed to proton beams or low‐energy photons restricts the straightforward application of EBT3 films. The relative effectiveness (RE) was introduced to quantify the LET quenching effect.^12^ In literature RE is mostly defined either as the ratio of the absorbed dose to water, $D_{w}$, and the film dose, $D_{\mathrm{film}}$, required to yield the same effect^7^, ^13^, ^14^, ^15^

(2) $$RE={\left.\frac{D_{\mathrm{film}}}{D_{w,Q}}\right|}_{\mathrm{iso}-\mathrm{effect}}=\frac{D(o_{Q_{0}}|p_{Q})}{D(o_{Q_{0}}|p_{Q_{0}})},$$

or as the ratio of $D_{w}$ and the apparent film dose, $D_{w,\mathrm{apparent}}$, applying the parameters obtained at $Q_{0}$ 5, 10, 12, 16, 17, 18

(3) $$RE={\left.\frac{D_{w,\mathrm{apparent}}}{D_{w,Q}}\right|}_{\mathrm{iso}-\mathrm{dose}}=\frac{D(o_{Q}|p_{Q_{0}})}{D(o_{Q_{0}}|p_{Q_{0}})}.$$

Those two definitions are fundamentally different and cannot be mutually exchanged due to the nonlinear dependency of dose and net optical density [Eq. (1)]. While the former definition is equivalent to the definition of relative biological effectiveness (RBE), it is experimentally more challenging to determine than the latter since $D_{\mathrm{film}}$ is a‐priori not known whereas $D_{w,\mathrm{apparent}}$ can be obtained trivially, hence, used throughout this work [Eq. (3)]. The reciprocal of RE can directly be used as a beam quality correction factor, $g_{Q,Q_{0}}$, to correct the apparent film dose in an experiment if defined as in Eq. (3).

The energy distribution of an initially mono‐energetic proton beam widens with increasing depth in the sample due to energy and range straggling. Due to nonelastic interactions with the targets’ nuclei secondary fragments such as secondary protons, helium or heavier ions appear in the radiation field. While secondary protons contribute considerably to the total dose, <2% of the total dose is contributed by ions with atomic numbers exceeding one. A beam quality in film dosimetry refers to the fluence distribution differential in energy of all ionizing particles within the active volume of the detector. The unrestricted LET is often encountered in proton therapy, which implies the simplification that delta electrons deposit their energy locally. As the LET effect response is generally ion type dependent, one often further simplifies to only considering primary and secondary protons, which is supported by the generally low‐dose contribution of helium and heavier ions.^19^, ^20^ Even after those two reductions of complexity of the particle field, the beam quality is determined by the proton fluence distribution differential in (unrestricted) LET, which depends on the depth, target material, initial beam energy, and the beam generating hardware. To assess the beam quality with a single number the LET distribution can be averaged weighted by the fluence or the dose deposition, in this work always referenced with $L_{t}$ and $L_{d}$, respectively. The term fluence‐averaged LET is commonly also referred to as track‐averaged LET as fluence may be calculated as the sum of track lengths divided by the volume.^21^

The LET quenching effect among the evolutions of EBT film versions appeared similar, which allows to translate findings from EBT or EBT2 films to EBT3 films.^14^, ^22^ Experiments to determine the RE can be grouped into experiments using quasi mono‐energetic beams or using beams with a mixed particle spectrum. The RE differential in energy can only directly be quantified in quasi mono‐energetic beams.^12^, ^14^, ^22^ One of those studies showed that the energy dependence is negligible for proton energies higher than 15 MeV and exhibits the highest variation at about 5 MeV and a peak around 1 MeV.^22^ In terms of electronic stopping power in water, $S_{w}$, this corresponds to 3.3, 7.9, and 26.1 keV/μm,^23^ respectively. Such low‐energy beams are typically not available in clinically dedicated facilities, which complicates the systematic investigation of the quenching differential in energy or LET. In a clinical environment the LET quenching occurs toward the end of the beam range, where the beam cannot be considered mono‐energetic. Consequently, the RE can directly be determined as a function of average beam quality^5^, ^17^ or depth.^24^, ^25^ A plethora of methods to correct the LET quenching was proposed in literature without one showing superiority to another. A fourth order polynomial correction based on the fluence average LET was applied to correct the depth dose distribution of a single energy (161.6 MeV) proton beam.^5^ Another model suggests a correction following the reciprocal of a linear increase with dose‐averaged LET, which was validated for three single energy beams (71.3 MeV, 71.3 MeV plus filter and 159.9 MeV) and one spread‐out Bragg peak (SOBP).^17^ In contrast to those two models based on the averaged LET, the model proposed by Fiorini et al. averages the correction factor differential in energy, hence, taking into account the local energy distribution.^22^

The purpose of this study was to characterize the differences and limitations of three averaging concepts. Dose‐ and fluence‐averaged LET were compared in terms of measured RE values within an SOBP, where the contribution of high and low LET protons was systematically altered. Additionally, a quenching correction method using the local proton LET distribution was developed and RE values extrapolated for a variety of beam arrangements in order to verify whether the dose‐averaged LET is a sufficient predictor in all tested scenarios.

## 2 MATERIALS AND METHODS

### 2.1 The $g_{Q,Q_{0}}$ models

The bimolecular model was used to correlate the net optical density, *o*, to absorbed dose to water, $D_{w}$:

(4) $$o\left(D\right)=o_{m}\frac{D_{w}^{a}}{D_{1/2}^{a}+D_{w}^{a}}$$

with the three free parameters *a*, $D_{1/2}$ and $o_{m}$, which need to be determined for each batch of films individually.

#### 2.1.1 Calculating $g_{Q,Q_{0}}$ from the particle energy fluence spectrum

Assuming the analytic relation between the net optical density and absorbed dose is known [Eq. (1)], the fundamental theorem of calculus can be used to calculate the net optical density from an energy spectrum using $dD\;=\;\Phi_{E}S\left(E\right)\rho^{-1}dE$.

(5) $$o=\int_{0}^{E_{\max}}\Phi_{E}S_{a}\left(E\right)\rho_{a}^{-1}f^{\prime }\left(E|{\vec{p}}_{q}\right)dE.$$

Hence, the electronic stopping power, $S_{a}\left(E\right)$, and mass density, $\rho_{a}^{-1}$, of the active layer of the EBT3 films, the particle fluence differential in energy, $\Phi_{E}\;=\;\frac{d\Phi }{dE}$, and the derivative of the calibration function at all energies, $f^{\prime }\left(E|{\vec{p}}_{q}\right)$, of a mono‐energetic beam quality, *q*, are required to calculate the net optical density. The former two quantities can be determined using for example Monte Carlo (MC) particle transport simulations. However, the calibration function at any mono‐energetic beam quality remains unknown and the energy dependence of the parameters of the calibration function needs to be determined. In this study, the bimolecular model [Eq. (4)] was used as a calibration function and the energy dependence shall be described with an energy‐dependent $D_{1/2}$ parameter (while keeping the parameters *a* and $o_{m}$ constant) using

(6) $$D_{1/2}=c_{1}+c_{2}\left(1-e^{-S_{w}^{2}{\left(2c_{3}\right)}^{-2}}\right),$$

with three parameters, $p_{Q}\;=\;\left\{c_{1},c_{2},c_{3}\right\}$, which were determined by inverse optimization as explained in the following section. Eq. (6) was chosen since it fits the analytic relationship $g_{q,Q_{0}}$ reported in literature (fig. 3 in Ref. [22]). The beam quality correction factor, $g_{q,Q_{0}}$, was converted to $D_{1/2}$ using the relationship $k\;=\;aD_{1/2}^{-a}$ in the formalism of the bimolecular model assuming *k* is proportional to $g_{q,Q_{0}}$ (see the derivation of eq. (4) in Ref. [5]), which were then fitted in order to obtain initial guess parameters $c_{1}^{0},c_{2}^{0},c_{3}^{0}$.

For numeric computation all formulas were evaluated as a function of stopping power in water, $S_{w}$, instead of energy to reduce computational effort. According to literature^22^ the LET quenching is negligible for proton energies above 15 MeV, while there is a steep gradient at very low energies (≈1 − 5 MeV). Therefore, a small energy binning at low energies is required, whereas a rougher energy grid is sufficient from 15 to 250 MeV. The stopping power in water ranges from 0.4 to 3.3 keV/μm in that energy regimen where no quenching is expected, whereas it increases from 7.9 to 26.1 keV/μm in the region with highest expected variations.^23^ Consequently, the computation time could be reduced substantially by using $S_{w}$ instead of *E* without the necessity of nonlinear energy binning. The $S_{w}$ has a maximum at around 0.08 MeV,^23^ hence, protons with lower energy than that are indirectly assumed to have the same quenching effect as a proton of higher energy exhibiting the same $S_{w}$. However, their contribution is negligible as <0.3% of the dose was contributed by protons with an energy lower than 0.08 MeV in all our simulations in water.

With Eq. (6) all variables are now known in order to solve the integral in Eq. (5) and calculate the net optical density from a given fluence distribution. As the integral needs to be solved numerically, we put this together in discrete form

(7) $$\delta o=o_{m}a\frac{1}{c_{1}+c_{2}\left(1-e^{-S_{w}^{2}{\left(2c_{3}\right)}^{-2}}\right)}{\left(\frac{o_{m}-o}{o_{m}}\right)}^{2}{\left(\frac{o}{o_{m}-o}\right)}^{1-1/a}\delta D.$$

Equation (7) is a numerical update rule of the net optical density at each iteration ($o_{i+1}\;=\;o_{i}\;+\;\delta o_{i}$) given a discrete dose deposition, *δD*, and can simply be derived from d*D*/d*o* of the inverse of Eq. (4). In our implementation we found $\delta D\;=\;5\cdot 10^{-5}$ Gy to be sufficiently small. The energy deposition was used instead of the product of fluence and stopping power as it is accessible more easily than the fluence in Geant4. Equation (7) has five parameters, $o_{m}$ and *a*, known from Eq. (4), and $c_{1}$, $c_{2}$ and $c_{3}$ replacing $D_{1/2}$ in order to account for the $S_{w}$ dependence. Those parameters can be derived from an optimization as explained below.

The apparent dose derived from the measured or calculated, $o_{Q}^{\mathrm{meas},\mathrm{calc}}$, at beam quality, *Q*, when applying the parameters obtained at a different beam quality, $p_{Q_{0}}$, was calculated from the inverse of Eq. (4). Subsequently, the apparent dose was used to calculate RE and $g_{Q,Q_{0}}$ according to Eq. (3).

The parameters $c_{1},c_{2},c_{3}$ and $o_{m}$ in Eq. (7) were determined by minimizing the cost function

(8) $$X^{2}=\Sigma_{i\in T}{\left(g_{Q,Q_{0}}^{i,calc}-g_{Q,Q_{0}}^{i,meas}\right)}^{2},$$

which is the sum of squared differences of all calculated and measured $g_{Q,Q_{0}}$ in the training data set, *T*, which include the calibration at 179 MeV, $Q_{0}$, and measurement of a SOBP (’$b_{1}$’) in water as explained in more detail in Section 2.B. The parameter *a* = 0.82 was adopted from literature to reduce the number of fit parameters.^5^

#### 2.1.2 Correlating $g_{Q,Q_{0}}$ to an averaged beam quality

In the simple $g_{Q,Q_{0}}$ model, RE was approximated by a first,

(9) $$RE\left(L_{d}\right)=a_{0}+a_{1}L_{d},$$

or fourth order polynomial of $L_{d}$ similar to Ref. [17]. As mentioned earlier, the correction factor, $g_{Q,Q_{0}}$, is the reciprocal of RE.

### 2.2 Monte Carlo simulations

Gate v8.0 in combination with Geant4.10.3.p01 was used to simulate the dose deposition, fluence spectra, $L_{t}$ and $L_{d}$. A validated beam model^26^, ^27^ was applied using the binary cascade model for inelastic nuclear interactions via the nuclear physics builder *QGSP_BIC* and the WentzelIV model for electromagnetic interactions (option 4, EMZ).

The *ComputeElectronicDEDX* function of the *G4EMCalculator* class in Geant4 was used to retrieve the unrestricted electronic stopping power and to calculate the averaged LET ($L_{d}$ and $L_{t}$) to assure an independence of step limiting events such as geometrical boundaries or a fixed step limiter.^28^, ^29^ The $L_{d}$ scoring method was equivalent and benchmarked to “Method C” in Ref. [28]. The fluence spectra were scored using the EnergySpectrumActor in Gate with an equidistant *S* bin size width equal to 0.05 keV/μm considering only ions with charge +1 (protons, deuterons, and tritons). Contributions of heavier particles were neglected due to its overall small contribution to dose.

The step limiter was set to 10 μm, the production cut for secondary protons to 5 μm in order to enable production of low‐energy protons. The default cut value is 0.7 mm, which corresponds to a 7.5 MeV or 5.7 keV/μm proton in water. Production of secondary $e^{-}$, $e^{+}$ and *γ* was inhibited by a cut value equal to 100 m. G4_WATER was used as material in the simulations with a mean excitation potential of 78 eV.

### 2.3 Measurements

All measurements were carried out at the nonclinical horizontal beamline (IR1) at the MedAustron Ion Therapy Centre. The synchrotron‐based facility provides pencil beam scanning and 255 actively selected energies ranging from 62.4 to 252.7 MeV, while lower energies could be achieved passively by inserting a range shifter (RaShi) of 3 cm PMMA.^30^ Five sheets of Gafchromic EBT3 (Lot#: 06291702) films were cut into rectangular pieces of 2.5 × 3.5 cm and scanned at the center of an Epson 11000 XL flatbed scanner (EPSON GmbH, Meerbusch, Germany) in transmission mode and portrait orientation. The net optical density was defined as the optical density of the sample before/after irradiation subtracted by the background, that is, the optical density of unirradiated film samples that underwent the same procedure as the irradiated samples. A circle with the same dimensions as the active area (diameter 15.6 mm) of the Roos ionization chamber (TM34001, PTW, Freiburg, Germany) was evaluated, which was much smaller than the lateral field size of 7 × 7 cm. Films were scanned before and after irradiation, sufficient preheating of the scanner was ensured by several empty scans. All experiments were carried out within 8 h and read out 48 h after irradiations within approximately 6 h in the same order as the irradiations. The film handling and readout procedure were performed as described in Refs. [31, 32]. Each film measurement represents the average of three repetitions (three films each scanned once). The water equivalent thickness (WET) of an EBT3 film was measured to be 0.358 mm at a nominal proton energy equal to 97.4 MeV. The energy dependence of the WET was neglected as it is expected to be much lower than the positioning accuracy. The reference point of each film was at the center of the active volume calculated applying a constant WET.

All ionization chamber dose measurements were carried out using a Roos IC (TM34001, PTW). The effective point of measurement was defined at the surface of the entrance window in the air cavity. The WET of the Roos chamber’s entrance window was also assumed to be independent of energy and equal to 1.3 mm. Absorbed dose to water was determined following IAEA TRS‐398 adapted to scanned ion beams.^33^ For calibration of the film net optical density to absorbed dose to water, that is, to retrieve the fit parameters in Eq. (4), two sets of measurements were carried out. First, the net optical density at 11 dose levels ranging from 0 to 10 Gy was determined in the entrance plateau region (at 2 cm depth) of a 179.7 MeV initial energy beam in water. At this high energy and position, the dose‐averaged LET is low (0.5 keV/μm considering primary protons only and 0.9 keV/μm including secondary protons), the dose and LET gradient are minimal and charged particle equilibrium is reached. A second set was measured in the highest possible $L_{d}$ region (4 keV/μm to 12 keV/μm) within a low‐dose gradient, a spread‐out Bragg peak in a shallow depth ranging from 3.0 to 3.5 cm in water, referred to as configuration “$b_{1}$.” This beam was composed of the lowest six available proton energies ranging from 62.4 to 67.5 MeV. Higher energy beams exhibit a higher energy spread toward the end of range resulting in a lower $L_{d}$ gradient. The weighting factor of each energy layer was derived from a least squares fit using MC simulated depth dose distributions (0.1 mm resolution in depth) and a desired dose level of 1 Gy.^34^, ^35^

In order to identify the superior single‐parameter averaging concept (fluence or dose weighting), the dose of the SOBP “$b_{1}$” was characterized with films at 16 depths all located within the SOBP. The 16 positions were measured in three separate irradiations using two stacks of five films and one stack of six films. The same measurement was repeated where 50% of the initial fluence of the previous configuration was replaced by the highest available energy beam (252.7 MeV); this experiment was labeled with “$b_{2}$.” Note that simply replacing 50% of the initial “$b_{1}$” fluence with the 252.7 MeV beam would result in a considerably lower dose in the SOBP plateau due to the lower stopping power. In order to remain at a constant dose in the SOBP plateau the total fluence had to be scaled by a factor 1.7. The 252.7 MeV beam contributed approximately 13% to the total dose in the dose plateau of the SOBP in “$b_{2}$.” In the third experiment of this series (“$b_{3}$”), 50% of the dose in the SOBP was replaced by the high‐energy beam (252.7 MeV). In terms of initial particle fluence this corresponds to 87% of the total fluence. There, the initial particle fluence was increased by a factor 3.4 in order to maintain a constant dose in the SOBP. In all three configurations the ratio of the initial beam weights of the low‐energy beams (62.4–67.5 MeV) remained constant.

**Figure 1:**
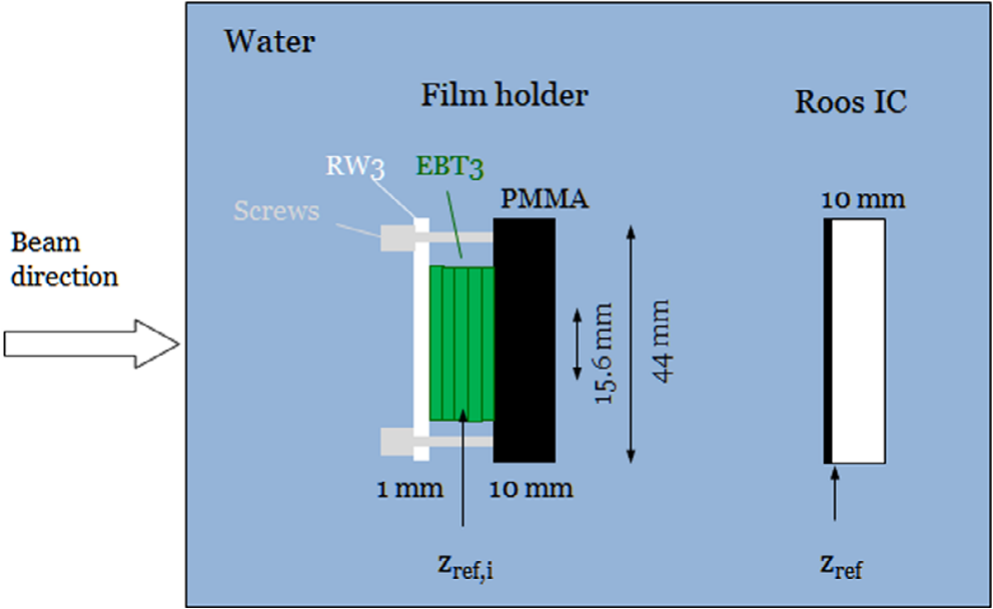
Schematic overview of the measurement setup in water. The custom film holder fits into the same Trufix Holder (PTW) as the Roos IC. IC and film measurements were carried out sequentially. [Color figure can be viewed at wileyonlinelibrary.com]

Figure 1 shows an overview of the measurement setup in water. The entrance window of the customized film holder was made of RW3 and 1 mm thick. The position of each film was corrected by the WET of all nonwater materials in the beam path. The WET of six films was approximately 2.1 mm. The film holder was manufactured such that it could be mounted in the Trufix holder (PTW, Germany) for the Roos IC. A remotely controlled water phantom (MP3‐PL, PTW) was used to assure accurate positioning and a minimum residence time in water. The water resistance of EBT3 films was confirmed by putting five cut pieces of EBT3 films into water. Even after 24 h the water penetrated <5 mm into the active area at the edges. To avoid a potential bias due to penetrating water, residual time of films in water was minimized in the measurements (<3 min) and film calibration was performed under identical conditions.^36^

### 2.4 Parameter estimation and validation

In order to determine the film dose $D_{w,\mathrm{apparent}}$ [the inverse of Eq. (4)] from a measured or calculated net optical density, the calibration parameters, $p_{Q_{0}}\;=\;\left\{o_{m},D_{1/2},a\right\}$, were fitted at reference conditions with beam quality $Q_{0}$ resulting in $p_{Q_{0}}\;=\;\left\{0.93,8.99,0.82\right\}$. The reference beam quality was the particle fluence at 2 cm depth in water of a 179 MeV beam, consisting mostly of low LET (less than 1 keV/μm) protons and a few percent of secondary particles exhibiting higher LET. The parameters were fitted in a dose range of 0 to 10 Gy, each dose level measured with the Roos IC. For all subsequent evaluations, the Roos IC measurements were only used to normalize the MC simulated dose, which was used as absorbed dose to water, $D_{w,Q}$, to calculate $g_{Q,Q_{0}}^{\mathrm{meas}}$ and *RE* according to Eq. (3). The MC simulated dose was assumed to be sufficiently accurate since it agreed better than 0.2% with the measured dose at all positions in the SOBPs in water (“$b_{1}$”, “$b_{2}$” and “$b_{3}$”), hence, the contribution to the total uncertainty was neglected.

To numerically calculate the net optical density according to Eq. (7), four parameters ($o_{m},c_{1},c_{2},c_{3}$) needed to be determined (the fifth parameter was adopted from literature), while the proton spectrum was known from MC simulations at each position. Starting from the initial guess parameters, the fit parameters were updated until the squared differences of the measured and calculated $g_{Q,Q_{0}}$ [Eq. (8)] reached a minimum using *fminunc()* in MatlabR2016b. The film measurements at reference conditions (calibration at 2 cm in 179 MeV beam) and the SOBP (“$b_{1}$”) served as training data set using only doses <2.5 Gy. The measured $g_{Q,Q_{0}}^{\mathrm{meas}}$ of the two beams, having 50% fluence (“$b_{2}$”) or 50% of the dose (“$b_{3}$”) contributed by the low LET beam, were used to validate the calculation method as their local particle spectrum is considerably different. All measurements including the calibration were carried out in water to assure high positioning accuracy and to avoid any systematic bias due to particle fluence perturbations originating in different materials.

In a next step $g_{Q,Q_{0}}^{\mathrm{calc}}$ values were calculated at 100 positions for nine different beam configurations according to Eq. (7) and the parameters obtained as described in the previous paragraph. The particle spectrum at each position was obtained from Monte Carlo simulations using the validated beam model. This comprehensive calculated data set was used to verify whether the averaged beam quality $L_{d}$ is sufficient to describe the $g_{Q,Q_{0}}$ (or the reciprocal value RE) in all those cases.

A simple correction function was derived applying a first and a fourth order polynomial fit of RE over $L_{d}$ on the calculated RE data set using Eq. (9). Since clinically relevant $L_{d}$ values are typically below 10 keV/μm, a 15 keV/μm upper limit was introduced in the fit. Four single energy beams (62, 148, 179 and 252 MeV), the three beams simulating the extreme multifield cases (“$b_{1}$”, “$b_{2}$” and “$b_{3}$”) and two cubic shaped SOBPs (modulation width 5 cm) generated with the Raystation TPS (single beam) centered at a shallow and deep depth (6 and 30 cm) were simulated.

Finally, the RE was evaluated as a function of dose (“$b_{1}$”) to determine the dose range of similar behavior motivating the choice of only considering doses <2.5 Gy.

An overall film positioning repeatability of ≤0.3 mm was found in other film experiments using the same measurement setup in‐house. For consistency with literature^17^ a value equal to 0.25 mm was propagated into the RE confidence interval (CI) calculation in addition to the standard deviation of the three film measurements.

## 3 RESULTS

### 3.1 Measurements

**Figure 2:**
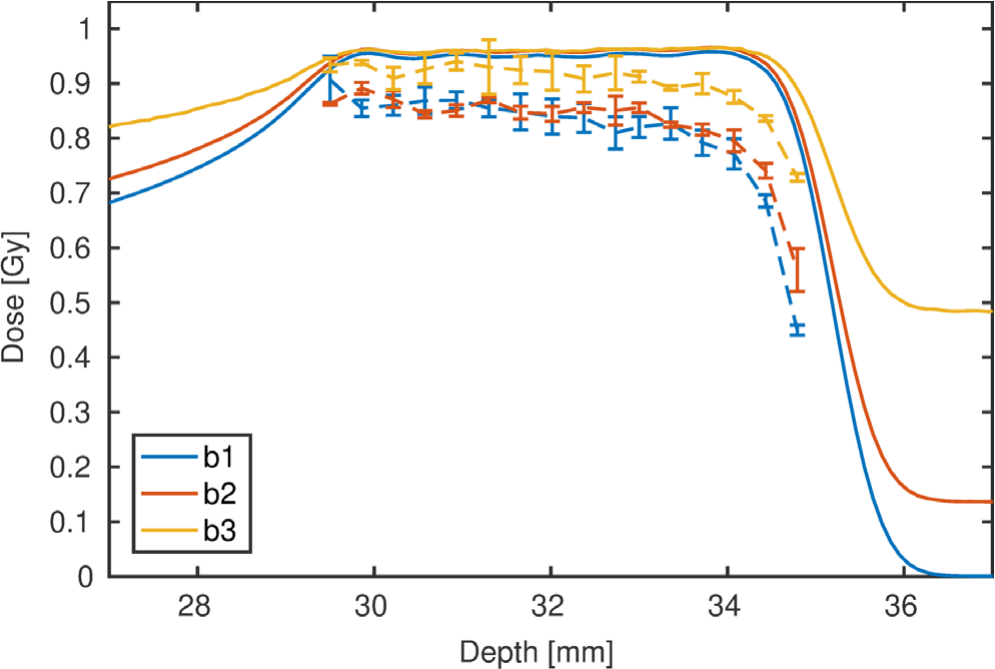
Uncorrected film dose (dashed lines) and Monte Carlo simulated dose (solid lines) of three spread‐out Bragg peak (SOBP) beams in water. The 50% and 87% initial fluence contribution of the high‐energy beam to the SOBP of “$b_{1}$” appears as a 0.1 and 0.5 Gy dose tail in “$b_{2}$” and “$b_{3}$,” respectively. The error bars represent the 1*σ* standard deviation of the three film measurements. [Color figure can be viewed at wileyonlinelibrary.com]

The measured uncorrected film dose using the calibration obtained at a low LET beam $p_{Q_{0}}$ and the MC simulated dose of the experiments in water are plotted in Fig. 2. The uncorrected film dose, $D_{w,\mathrm{apparent}}$, underestimated the dose plateau by 2 to 26% excluding the most distal measurement point. The distal measurement point was excluded also in the following due to the steep dose gradient and the associated high uncertainties.

### 3.2 Comparing fluence‐ and dose‐averaged LET

**Figure 3:**
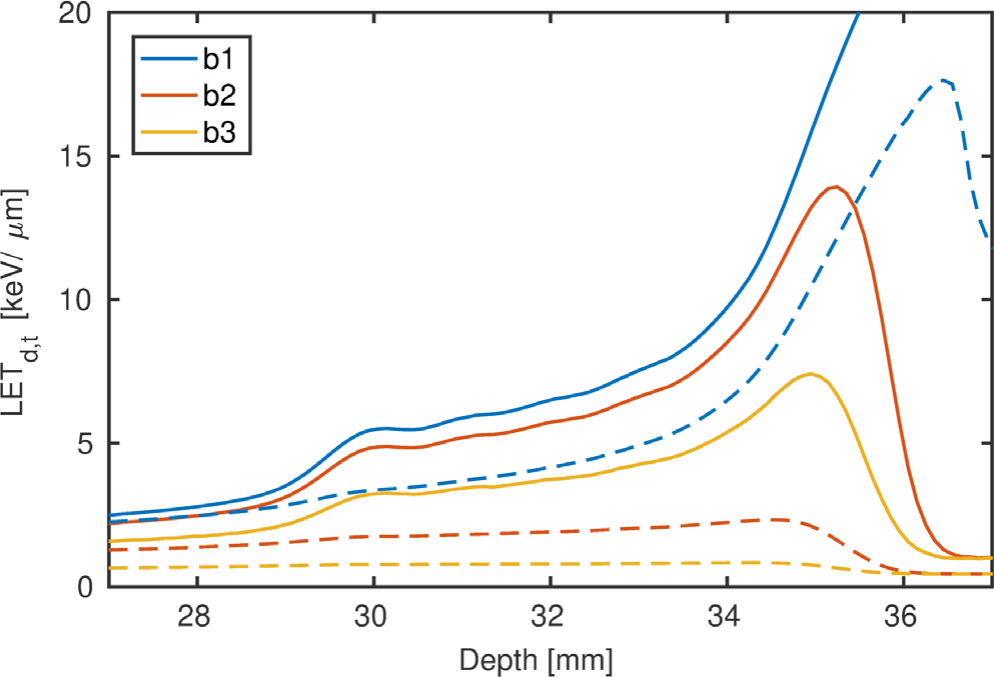
Monte Carlo simulated $L_{d}$ (solid lines) and $L_{t}$ (dashed lines) of “$b_{1}$,” “$b_{2}$” and “$b_{3}$” in water. [Color figure can be viewed at wileyonlinelibrary.com]

The $L_{d}$ and $L_{t}$ distribution over depth of the three beam arrangements with varying energy distribution are shown in Fig. 3. The $L_{d}$ at the measured film positions ranged from 4.5 to 14.5 keV/μm, 4.0 to 12.3 keV/μm, and 2.7 to 7.3 keV/μm for beams “$b_{1}$” to “$b_{3}$”, respectively. This corresponds to an increase by a factor of 3.2, 3.0 and 2.7, respectively. While $L_{d}$ increased strongly with depth in all beams, $L_{t}$ hardly increased for beams “$b_{2}$” and “$b_{3}$.” From the beginning to the end of the SOBP $L_{t}$ only increased by 35% and 5% in “$b_{2}$” and “$b_{3}$.” This modest increase in $L_{t}$ with depth cannot correlate the decrease in the relative effectiveness of the films with depth. The determined film doses of “$b_{1}$” and “$b_{2}$” plotted in Fig. 2 were within the precision, while the $L_{t}$ distribution (see Fig. 3) differed substantially.

**Figure 4:**
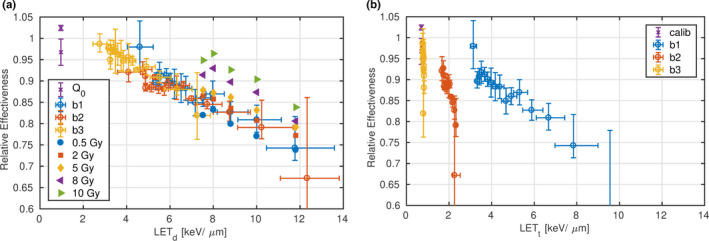
Relative effectiveness as a function of dose‐ (a) and fluence‐ (b) averaged linear energy transfer. relative effectiveness (RE) measured at a constant dose equal to 1Gy are represented with open markers and error bars. The dose and $L_{d}$/$L_{t}$ confidence interval is derived from the positioning uncertainty. The former inherits in addition the standard deviation of the three repeated irradiations. The RE at varying dose levels measured in the distal part of “$b_{1}$” are illustrated with filled symbols (a). [Color figure can be viewed at wileyonlinelibrary.com]

The REs of “$b_{1}$,” “$b_{2}$” and “$b_{3}$” are expressed as a function of $L_{d}$ and $L_{t}$ in Figs. 4(a) and 4(b), respectively. At a constant dose (1 Gy) the RE of “$b_{1}$,” “$b_{2}$” and “$b_{3}$” decreased approximately linearly with $L_{d}$. In contrast to the one‐to‐one relation of RE and $L_{d}$, no functional relationship between RE and $L_{t}$ could be observed encompassing all beam arrangements.

The residuals of the calibration function at reference conditions are included in Fig. 4 for 0.75 and 1 Gy to illustrate the approximate 3% systematic dose uncertainty due to the calibration function. The deepest measurement point of “$b_{1}$” to “$b_{3}$” was excluded in all analysis due to the high local dose and $L_{d}$ gradient, which makes this point prone to positioning uncertainty. Nevertheless, this last point reflects characteristics of $L_{t}$ in “$b_{2}$” and “$b_{3}$.” The peak of $L_{t}$ is pushed back with increasing contribution of low LET protons (252.7 MeV). The $L_{t}$ ($L_{d}$) peak positions were 34.5 mm (35.3 mm) and 34.3 mm (35.0 mm) for “$b_{2}$” and “$b_{3}$,” respectively. As a result, $L_{t}$ at the last measurement point (34.8 mm) was lower than at the penultimate position (34.4 mm), while the RE continued to decrease. The high RE uncertainty at that point was dominated by the dose gradient, while the standard deviation of the three repeated measurements was similar to the other measurements (see Fig. 2).

### 3.3 Quenching correction based on energy spectrum

**Figure 5:**
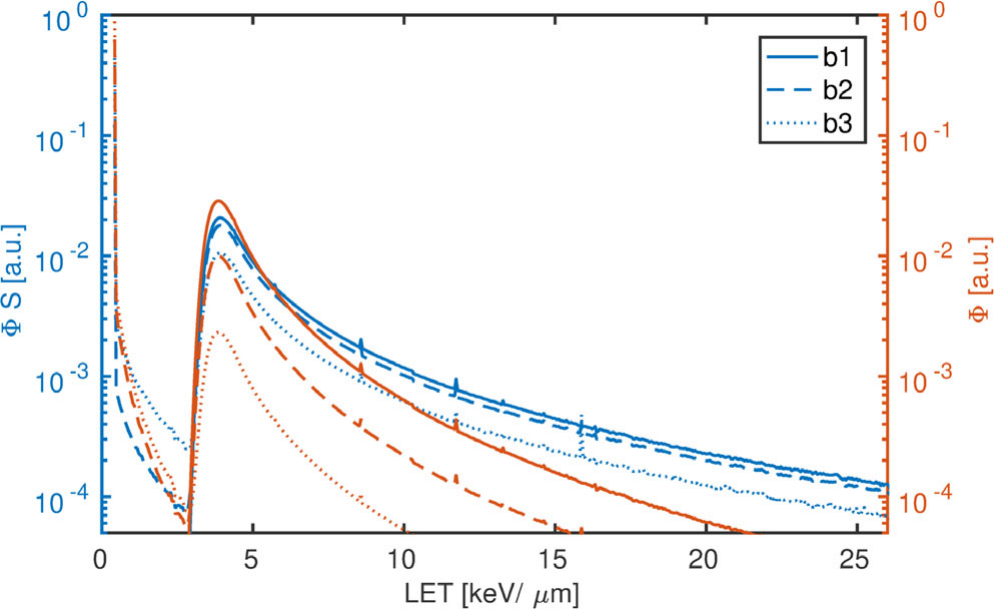
Simulated energy spectra of “$b_{1}$” (solid line), “$b_{2}$” (dashed line) and “$b_{3}$” (dotted line) in water at a constant position. The energy deposition is plotted against the left vertical axis in blue, while the fluence is plotted against the right axis in red. All curves are normalized to the integral over the entire spectrum for better visualization. [Color figure can be viewed at wileyonlinelibrary.com]

At each film position the proton energy spectrum was simulated using Gate/Geant4; in Fig. 5 the energy deposition (Φ*S*) and fluence Φ histograms at 33.4 mm are shown. At this position the LET distribution of “$b_{1}$” starts at 3 keV/μm and reaches a maximum around 4 keV/μm. The additional contribution of the high‐energy beam can be observed as a global sharp peak at 0.4 keV/μm in “$b_{2}$” and “$b_{3}$” resulting in a nonunimodal LET distribution. To describe those beam qualities with a single number one may use the dose‐averaged LET, which was 8.2, 7.2, and 4.6 keV/μm for “$b_{1}$,” “$b_{2}$,” and “$b_{3}$,” respectively.

**Figure 6:**
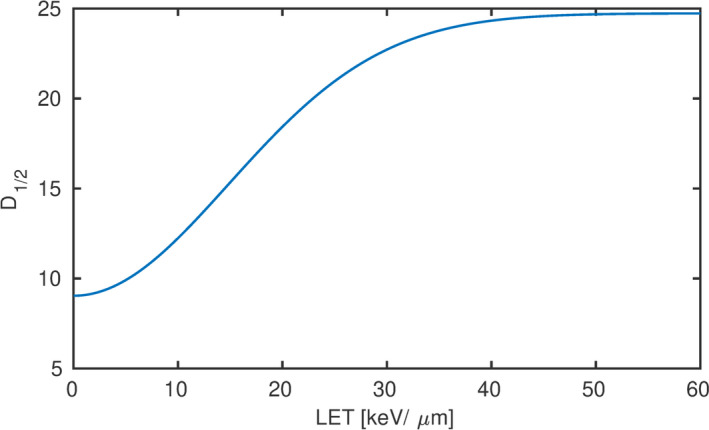
$D_{1/2}$ as a function of linear energy transfer resulting from minimizing the squared differences of the measured and calculated $g_{Q,Q_{0}}$. [Color figure can be viewed at wileyonlinelibrary.com]

The net optical density measured in “$b_{1}$” and in the calibration (179  MeV), the MC simulated LET spectra and dose were used to determine $D_{1/2}$ applying the $X^{2}$ minimization [Eq. (8)]. This optimization resulted in parameters $o_{m}\;=\;0.96$, *a* = 0.82, $c_{1}\;=\;9.0$, $c_{2}\;=\;15.7$, $c_{3}\;=\;14.8$ [Eq. (6)], which gives the $D_{1/2}$ as a function of LET plotted in Fig. 6. $D_{1/2}$ increased moderately (up to 10%) from 0 to 5 keV/μm, followed by a steep increase, which started to flatten out at around 30 to 40 keV/μm.

**Figure 7:**
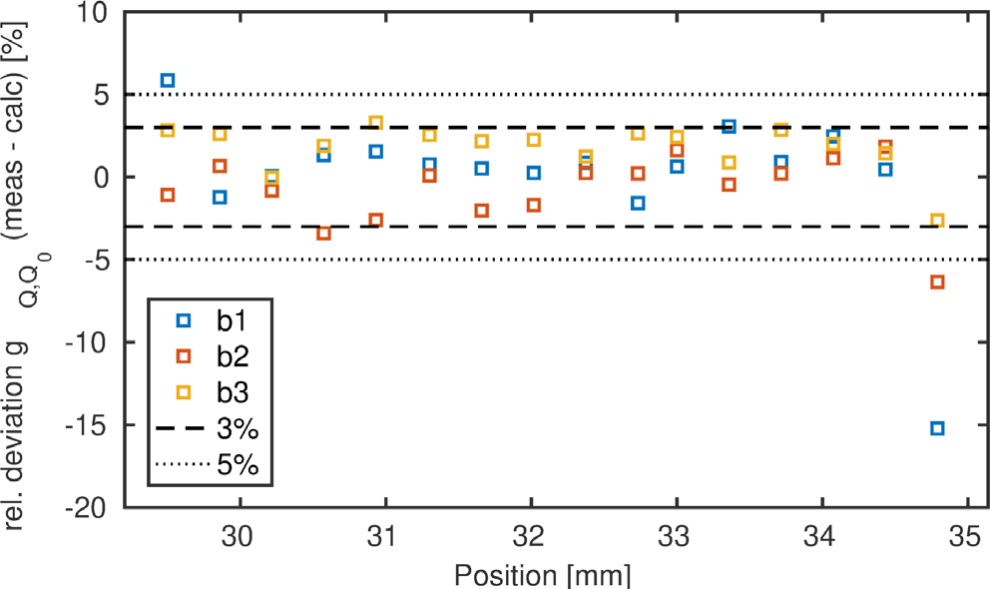
Relative deviation of the calculated to the measured $g_{Q,Q_{0}}$ correction factors over depth. [Color figure can be viewed at wileyonlinelibrary.com]

Using the optimized relationship of $D_{1/2}$ as a function of LET, $g_{Q,Q_{0}}$ can be calculated. Figure 7 shows the difference of calculated and measured $g_{Q,Q_{0}}$ factors. Negligible differences were found for the training data (“$b_{1}$”), which were used for fitting, indicating that the optimization converged. The two beams with increasing contribution of low LET protons (“$b_{2}$” and “$b_{3}$”) were not included in the fitting procedure but served as validation set. The $g_{Q,Q_{0}}$ calculated from the energy spectra agreed within 3% with measurements for “$b_{2}$” and “$b_{3}$.”

### 3.4 Extrapolation of REs

**Figure 8:**
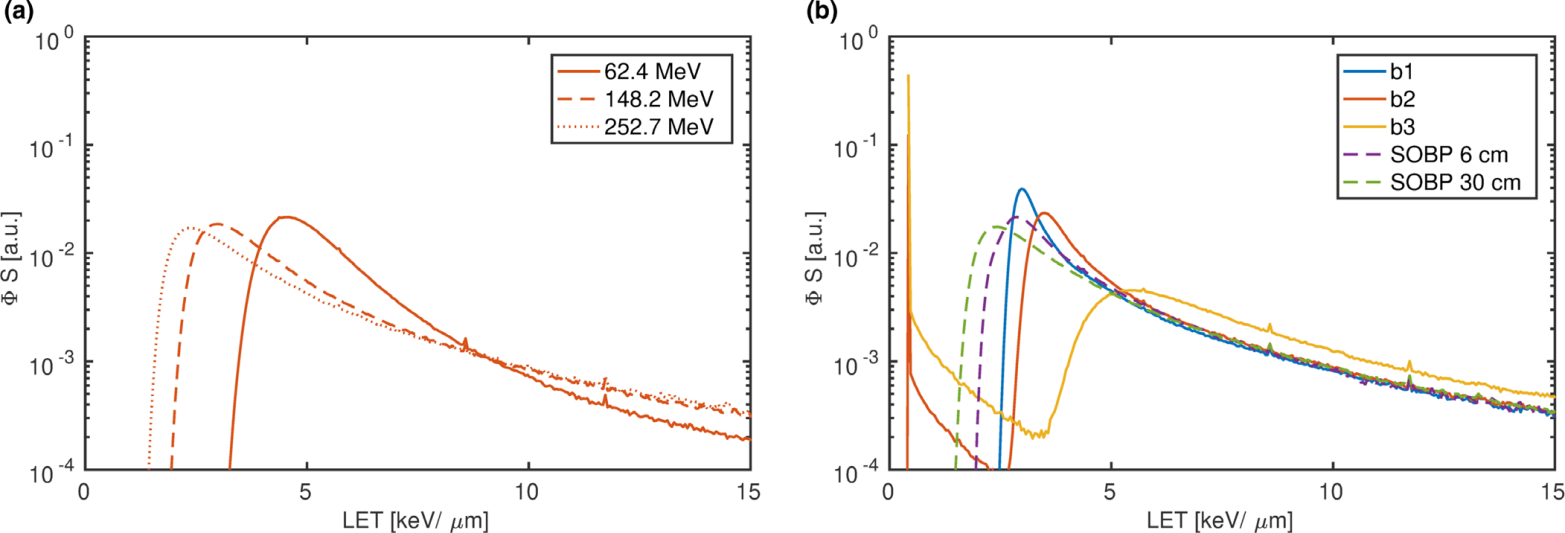
Simulated dose deposition spectra of protons differential in linear energy transfer yielding an iso‐$L_{d}$ equal to 6.4 keV/μm. Single energy beams are illustrated in (a). Box shaped spread‐out Bragg peaks with a side length of 5 cm centered at 6 and 30 cm are plotted together with beams “$b_{1}$,” “$b_{2}$” and “$b_{3}$” in (b). [Color figure can be viewed at wileyonlinelibrary.com]

**Figure 9:**
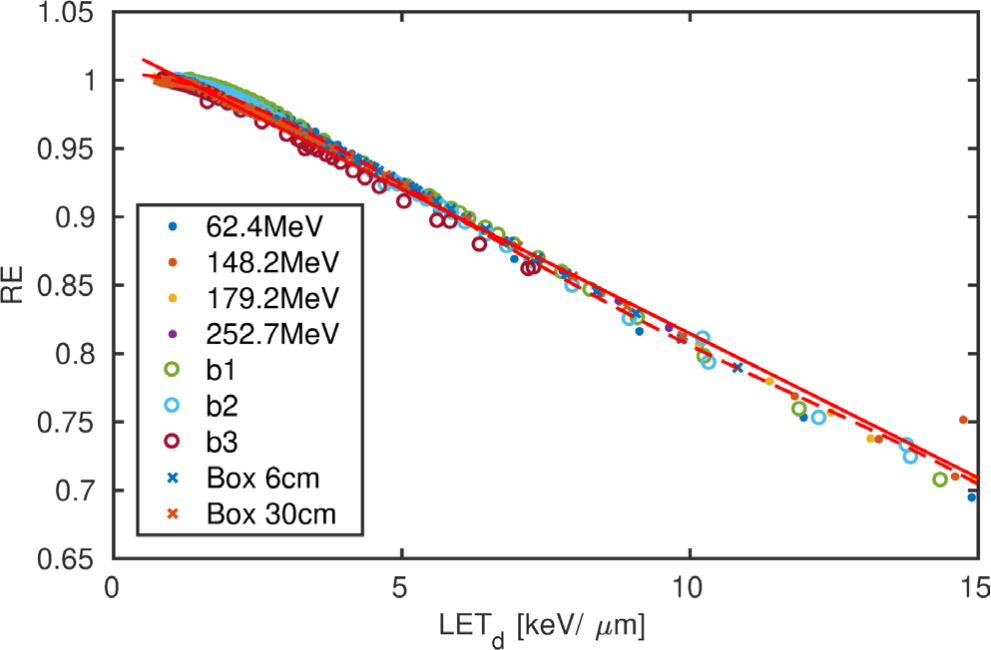
Calculated relative effectiveness as a function of dose‐averaged linear energy transfer for four single energy layers, the beams “$b_{1}$”–“$b_{3}$“ and two cases obtained from a commercial TPS centered at 6 and 30 cm depth. Relative effectiveness (REs) of all cases (at constant $L_{d}$) are almost overlaying, indicating that RE correlates with $L_{d}$ in all nine beam configurations. The first and fourth order polynomial fits are represented with solid and dashed lines, respectively. [Color figure can be viewed at wileyonlinelibrary.com]

Figure 8 shows the LET distribution of protons in eight different beam arrangements resulting in an iso‐$L_{d}$ equal to 6.4 keV/μm. Although the dose weighted average of the distribution resulted in the same value, the distributions differed substantially. The mode (peak position) of the LET distribution of single energy beams decreased from 4.6, 2.9 to 2.5 keV/μm with initial beam energies increasing from 62.4, 148.2 to 252.7 MeV, respectively, due to the increasing dose contribution of high LET protons. While protons with an LET ranging from 1.4 to 3.2 keV/μm account for 42% of the dose deposition in the highest energy beam, they are negligible in the lowest energy beam (51 ppm dose contribution). The decrease in the mode (2.9 to 2.5 keV/μm) and widening of the LET distribution could also be observed for the SOBP plan composed of higher initial energies and centered at 30 cm compared to the SOBP plan at 6 cm depth. The LET distribution of the iso‐$L_{d}$ position in “$b_{1}$” was of similar shape as the before mentioned, exhibiting a single mode at 3.0 keV/μm and a negligible dose contribution of protons below an LET threshold of 2.5 keV/μm. However, the LET distribution of “$b_{2}$” and “$b_{3}$” differed considerably. A global mode at 0.4 keV/μm appeared due to the superposition of the 252.7 MeV beam. Protons with an LET below 2.5 keV/μm contributed 13% and 48% to the total dose in “$b_{2}$” and “$b_{3}$,” respectively. The local modes increased from 3.5 and 5.5 keV/μm with the increasing dose contribution of the low LET protons of “$b_{2}$” and “$b_{3}$,” respectively. Despite the considerably different LET distributions of the iso‐$L_{d}$, the calculated RE using the correction model differential in LET resulted in approximately the same RE. In Fig. 9 REs calculated from the simulated energy spectra for those nine beam arrangements are shown as a function of $L_{d}$.

The extrapolated REs decreased approximately linearly with $L_{d}$ for $L_{d}$ greater 2 keV/μm in all beams with a moderate shoulder from 1 to 2 keV/μm. A first and fourth order polynomial were fitted on those data, both fitting the data well. The fit resulted in parameters: $a_{0}\;=\;1.0258$, $a_{1}\;=\;-0.0211$ (keV/μm)${}^{-1}$ and $b_{0}\;=\;1.0054$, $b_{1}\;=\;-6.4262\cdot 10^{-4}$ (keV/μm)${}^{-1}$, $b_{2}\;=\;-4.9426\cdot 10^{-3}$ (keV/μm)${}^{-2}$, $b_{3}\;=\;4.1747\cdot 10^{-4}$ (keV/μm)${}^{-3}$, $b_{4}\;=\;-1.1622\cdot 10^{-5}$ (keV/μm)${}^{-4}$, respectively. The REs of those two parametrizations differ by <1%. Therefore, only the linear function was applied to calculate $g_{Q,Q_{0}}$ in the following. Typical $L_{d}$ values of 1 (entrance plateau), 5 (distal region of a SOBP) and 10 keV/μm (falloff of a SOBP), hence, result in a RE equal to 1.0, 0.9, and 0.8, respectively.

### 3.5 Dose dependence

The filled markers in Fig. 4 show the RE of “$b_{1}$” at dose levels varying from 0.5 to 10 Gy. At constant $L_{d}$, the RE systematically increased with absorbed dose, or in other words, the LET quenching decreased with increasing dose. As the RE was comparable at doses up to 2 Gy, only doses lower than 2.5 Gy were considered. At $L_{d}$ lower 10 keV/μm, the RE was within −2% and +4% for the dose levels 0.5 and 2 Gy.

## 4 DISCUSSION

The measurement results of this study showed that $L_{t}$ could not be used as a single‐parameter radiation quality descriptor for all beam arrangements. Dose‐averaged LET, however, could describe the RE decrease in all three experimental beam setups. To provide evidence that $L_{d}$ also suffices in a more comprehensive data set, REs were calculated applying a novel formalism based on the local proton LET distribution. A formalism was proposed and validated to calculate the net optical density and beam quality correction factors from the proton LET spectrum. The dependence of the $D_{1/2}$ parameter on proton LET was derived from a least squares optimization of the differences between measured and calculated $g_{Q,Q_{0}}$ within a flat dose distribution. By applying this method, the dose deviations between films and MC simulations decreased from 26% to <3% in the simple as well as the more complex beam arrangements.

Another model to calculate beam quality correction factors based on the local particle spectrum was proposed by Fiorini et al.^22^ As it is based on a fluence average it was expected to fail for the “$b_{2}$”/“$b_{3}$” arrangement, but it also significantly underestimated the $g_{Q,Q_{0}}$ values of “$b_{1}$” measured within this study (see supplementary materials). This systematic deviation was also observed in an independent study.^37^ In our study, $g_{Q,Q_{0}}$ decreased with increasing dose [see Fig. 4(a)]. Consequently, a potentially higher dose level may explain the lower $g_{Q,Q_{0}}$ in Fiorini et al.^22^

Using an inverse optimization approach allowed to calibrate films differential in LET from experiments exhibiting a mixed LET field conducted at a clinical facility, which only provides rather high energies (typically above 60 MeV corresponding to 1 keV/μm in water). Usually experiments at low‐energy facilities are required to characterize films at higher LET^12^ implicating potential systematic biases. As films are sensitive to a variety of parameters, the different delivery characteristics, the environmental conditions within or on the way to the facility need to be considered. Additionally, low‐energy experiments are susceptible to positioning uncertainties and the energy variation within the detector. While the herein presented method is inherently less sensitive to those considerations, the disadvantage of the optimization approach is that it could result in a local minimum and it relies on MC simulations.

The bimolecular model was chosen for this study as it was applied in similar LET studies before.^5^, ^38^ For consistency, the exponent (in the notation of this study *a*, while *p* was used in literature; in this study *p* is reserved for a general parameter set) was fixed to 0.82. However, other functions than the bimolecular model, whose derivative is real and continuous in the relevant dose interval, could be used. As the specific form of Eq. (7) depends on the choice of the calibration function, the extrapolated RE may be model dependent. The experimental RE (Fig. 4), however, solely depend on the quality of the calibration at $Q_{0}$ and found to be model independent in our measurements. With respect to the repeatability of the measurements there was no significant difference in the accuracy of the fit to the calibration data at $Q_{0}$ using the bimolecular,^5^ the single channel^8^ or the gamma‐distributed single‐hit model.^7^

Although the analytical form of Eq. (5) is simple, its numerical implementation including the LET spectra calculation and data processing is time consuming. A linear relationship of RE and $L_{d}$ may be recommended for practical use as it approximated the artificial data well in all cases. The fit parameters of the linear function in this study were in good agreement to those derived by fitting 29 REs of three single energy beams reported in a recent study ($RE\;=\;1.02-0.0251\;L_{d}$).^17^ The RE of that function predicts up to 0.04 lower REs (at 9 keV/μm, the maximum $L_{d}$ in that study) compared to the function obtained here. Although the difference is within the uncertainty reported (7%, at 9.3 keV/μm), the different dose levels (2 Gy in the study published by Anderson et al., and 1Gy in this work) at which the RE were determined may explain the lower RE predicted in their study. Additional studies are required to investigate whether the $L_{d}$ correction is independent of film batch, scanner, or their combination.

An extreme case of clinical beams coming from different directions was mimicked with the “$b_{1}$”‐“$b_{3}$” experiment. A single directional SOBP with the lowest energy spread and hence highest $L_{d}$ deliverable was created (“$b_{1}$”). To experimentally identify whether the fluence or dose averaging concept is more appropriate, the energy/LET distribution was changed by applying a plan (“$b_{2}$”) where 50% of the initial particle fluence was replaced by highest energy/lowest LET protons deliverable in clinical mode and another plan where 50% of the dose in the SOBP was deposited by the low LET protons (“$b_{3}$”) while keeping the total dose constant. The experiments clearly show that dose averaging should be chosen over fluence averaging. Even for that extreme case, the simple correction on $L_{d}$ worked, whereas $L_{t}$ cannot be used for multifield plans. Such experiments may be recommended to other fields where the averaging concept is under discussion.

As shown in Fig. 5 averaging over the fluence underestimates the increased energy deposition of high‐LET/low‐energy protons. Hence, fluence averaging indirectly assumes a constant stopping power and a constant number of ionizations per proton independent of its energy.

$L_{d}$ to water was used since this should be available in all TPS and MC particle transport codes. The material composition of EBT3 films is not exactly known. The values reported in literature^5^, ^22^, ^38^ are inconsistent and contacting the vendor of EBT3 films did not result in a clarification. The SPR of the active material was reported to differ <2% from unity.^12^

Secondary particles heavier than protons are produced in nonelastic scattering events of the incident protons with the target nuclei. The dose contributed by those heavier secondaries is low, with alpha particles depositing most of the dose of heavier secondary ions. Depending on the initial beam energy up to ≈1% dose can be contributed by alpha particles.^19^, ^20^, ^39^, ^40^ Those heavier secondary ions were neglected in this study since, we assume that, the dosimeter (in combination with the protocol) used in this study was not sufficiently accurate and precise to enable investigation of such small dose contributions. Although the contribution of those heavier charged particles to absorbed dose is low, their LET is considerably higher than that of protons. Hence, including alpha particles in the $L_{d}$ calculation causes an increase by a factor of 2 to 3 in the entrance region.^40^ Averaging the stopping power over different ion types is equivalent to assuming that the effect correlates with stopping power independent of the ion type. There is insufficient knowledge of the LET quenching for other particles than protons to evaluate if this is the case. For the beam quality effect in other systems, for example the relative biological effectiveness (RBE) in cell cultures it is known that the RBE‐LET relation depends on the particle type.^41^, ^42^ An alternative to $L_{d}$ to quantify the beam quality in a mixed particle field was proposed for RBE^43^ and may also be tested for EBT3 films in future studies. The underresponse of EBT3 film dose in low‐energy photon beams may point to investigations of similarities in the dose deposition mechanisms and a beam quality descriptor beyond LET.^11^ One similarity could be the electron slowing down spectrum, which is of similar shape in a clinical proton beam compared to a medium‐energy x‐ray beam.^44^, ^45^

The proton LET distribution was obtained from MC simulations. Therefore, the results in our study depend on the accuracy of the GATE/Geant4 simulations. Thus, compatibility to other MC codes, Geant4 versions and settings has to be tested, in particular because the LET quenching appeared to be dominated by low‐energy protons in the single figure MeV region. The correction method based on $L_{d}$ was implemented into GATE and will be publicly available in the next release.

## 5 CONCLUSIONS

In this study a formalism was derived to calculate the net optical density and therefrom the $g_{Q,Q_{0}}$ for a specific EBT3 film batch from the local proton LET spectrum. Although the quenching effect was nonlinear as a function of LET, the RE could be well approximated by a linear function of the dose‐averaged LET in all tested beams. Both correction methods are restricted to proton beams in the clinically relevant energy range and dose ranging from approximately 0.5 to 2 Gy.

To provide experimental evidence whether fluence or dose weighting should be applied, the local energy distribution in a high LET SOBP was altered by superimposing a high‐energy (low LET) proton beam. The experiments demonstrated that fluence‐averaged LET cannot be used as a single‐parameter descriptor of the proton beam quality for radiochromic film dosimetry, whereas dose‐averaged LET was sufficient in all tested scenarios.

## ACKNOWLEDGMENTS

The authors thank our colleagues at the Medical University of Vienna and the MedAustron Ion Therapy Centre for their support, sharing data and helpful discussions, namely Ralf Dreindl, Natalia Kostiukhina, Fatima Padilla Cabal, Monika Clausen, Peter Kuess and Gerd Heilemann. Moreover, we thank Jose Baeza, Gabriel Paiva Fonseca, and Frank Verhaegen (Maastro Clinic, Netherlands) for sharing their triple‐channel read‐out implementation. The financial support by the (Austrian) Federal Ministry for Digital, Business and Enterprise and the National Foundation for Research, Technology and Development is gratefully acknowledged.

## Supporting information


**Figure. S1.** Measured vs. calculated beam quality correction factors using the Fiorini et al. 2014 model. The calculated factors are considerably lower than measured in the b1, b2 and b3 experiment.
